# Reprogrammable Hydroplastic Latex Films and Water‐Driven Phase Separation

**DOI:** 10.1002/advs.202523178

**Published:** 2025-12-27

**Authors:** Yuwei Du, Hanying Zhao

**Affiliations:** ^1^ College of Chemistry and Key Laboratory of Functional Polymer Materials of the Ministry of Education Nankai University Tianjin China

**Keywords:** hydroplastic latex film, latex particles, RAFT emulsion polymerization, water‐driven phase‐separation

## Abstract

Thermoplastics are a class of plastic polymers that can be remolded repeatedly by heating and cooling. Recently, inspired by the concept of thermoplastics, hydroplastic materials, a type of materials that can be reshaped with the aid of water, have aroused great interest. In this research, a simple and versatile approach to the preparation of hydroplastic latex film is developed and water‐driven phase separation in the film is studied. Core‐shell latex particles with protonated poly(2‐(dimethylamino)ethyl methacrylate) (*p*‐PDMAEMA) shells and poly(*n*‐butyl acrylate‐*co*‐methyl methacrylate) P(*n*BA‐*co*‐MMA) cores are synthesized, and latex films are prepared by solution casting. After water treatment, the films can be molded into various shapes and fixed into three dimensional shapes in dry state. The hydroplastic behavior is dependant on the phase structure. In the latex film there exist hydrophobic P(*n*BA‐*co*‐MMA) phases surrounded by continuous hydrophilic *p*‐PDMAEMA phases. In aqueous solution, water molecules diffuse into the hydrophilic phases, resulting in an increase in the volume ratio of the hydrophilic phases and a decrease in the mechanical properties; and the latex films are endowed with the hydroplasticity. The swollen of the hydrophilic phases by water allows the boundary interdiffusion of P(*n*BA‐*co*‐MMA) chains and the coalescence of the hydrophobic phases.

## Graphical abstract

Hydroplastic latex films with discontinuous hydrophobic phases surrounded by continuous hydrophilic phases, are prepared. Upon immersion in water, the latex films have water‐driven phase separation.

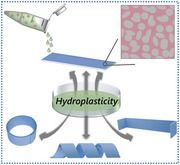

## 1 Introduction

Thermoplastic polymers are a class of linear or branched polymers that exhibit softening upon heating and hardening upon cooling within a specific temperature range [1, 2]. Thermoplastic polymers can be shaped under heating. Based on structures and physical properties, thermoplastic polymers can be classified into thermoplastics and thermoplastic elastomers (TPEs) [3, 4]. TPEs generally have two incompatible phases. The rigid phases act as junction points to give a crosslinked elastomer network and the soft phases provide elasticity [5, 6, 7, 8, 9, 10, 11, 12, 13, 14, 15]. Styrene‐butadiene‐styrene block copolymer (SBS), styrene‐ethylene‐butylene‐styrene block copolymer (SEBS), poly(butylene terephthalate) (PBT), thermoplastic polyamide elastomer (TPAE), and thermoplastic polyurethane (TPU) are typical TPEs [16]. TPEs have mechanical properties similar to vulcanized rubber, yet they are processable and widely used in coatings, adhesives, elastomers, fibers, and foams [17]. Global production of TPEs is expected to reach 5.55 million tons in 2026 [18].

TPEs have multi‐phase structures, and the phase structure changes upon heating in thermal processing. For example, Terban and coworkers studied the evolution of microphase separation of TPUs. They found that at an annealing temperature above 80^°^C the interdomain spacing increased significantly, suggesting that the mobilities of both soft and hard segments were required to achieve appreciable microphase segregation [19]. Wang and coworkers demonstrated that thermal annealing induced an increase in the spacing of hard and soft domains in TPEs [20]. Yuan and coworkers reported microphase separation of polyamide 6 (PA6)‐based TPEs in thermal treatment processes [21]. Other studies also indicated that thermal annealing promoted phase separation between the soft and hard phases in TPEs [22, 23, 24, 25]. Thermal annealing exerts two effects on TPEs: it facilitates the phase separation and imparts shaping capability to the materials.

The thermal processing techniques face some challenges, including expensive machines, harsh environmental conditions, and high energy consumption [26, 27, 28, 29, 30, 31]. Recently, researches on the synthesis of hydroplastic materials have aroused extensive attention due to the eco‐friendly and low‐cost processes. Materials that can be shaped and processed with the aid of water are referred to as hydroplastic materials. The principle of hydroplasticization is that the hydrophilic components in materials absorb water, and water molecules diffuse into the interiors breaking non‐covalent interactions such as hydrogen bonding and electrostatic interaction, making materials soft and moldable [32].

Biopolymers especially cellulose, have been widely used in the synthesis of hydroplastics. Zhang and coworkers made chemical modification on cellulose, and the modified cellulose membranes became soft and could be programmed into diverse 2D/3D shapes in the wet state [32, 33, 34, 35]. Zeng and coworkers prepared shape‐memory cellulosic hydroplastics by copper‐coordinated mercerization of nanocellulose paper [36]. Wu and coworkers prepared sustainable plastics by in‐situ polymerization of a monomer in the presence of hydroxypropyl methyl cellulose [37, 38]. Huang and coworkers fabricated hydroplastic polymers by constructing a dynamic dual cross‐linking network between cellulose nanofibers and synthetic copolymers [39]. He and coworkers fabricated cellulose hydroplastics by deacetylation of solution‐casted cellulose acetate sheets, and the materials can be shaped repeatedly into various 2D/3D geometries after being treated in water [40]. Wang and coworkers synthesized recyclable, flexible and degradable hydroplastics through the complexation of methylcellulose and tannic acid in water, followed by hot‐pressing [41]. Yan and coworkers prepared crosslinked networks by thiol‐ene and thiol‐oxazoline reactions [42], and hydroplastic materials were obtained after solvent evaporation and thermal treatment. Currently, the preparation of hydroplastic materials also faces challenges, including only a few options for precursor materials, organic solvent use, high temperatures in water treatment, and prolonged water treatment time. To expand the scope of the applications of the hydroplastic materials, it is necessary to find more precursor materials and develop novel eco‐friendly methods for the synthesis of hydroplastic materials.

Inspired by the structures of TPEs, we design hydroplastic latex films with two‐phase structures, one is hydrophilic and the other is hydrophobic phase. The hydrophobic phases serve as physical crosslinking sites, and the hydrophilic phases absorb water and endow the films with hydroplasticity. Efficient synthesis of hydroplastic latex film can be carried out without any organic solvent. Only water is used in the synthetic process. Similar to the thermal‐annealing phase separation in the thermoplastic materials, water‐driven phase separation was observed in the water treatment of hydroplastic latex film.

It is worthy of note that although multiphase hydrogels were reported previously [43, 44, 45], there are big differences between the two materials. The multiphase hydrogels have crosslinked three‐dimensional network structures, and the cross‐linking density is a key structural parameter. However, there are no crosslinking structures in the hydroplastic latex films, neither physical crosslinking structures nor covalently bonded network structures. The hydroplastic latex films absorb water in the hydrophilic phases, and the amount of water absorbed by the films is strongly dependent on the chemical composition of the hydrophilic polymers and the volume ratio of the hydrophilic phase in the film.

## 2 Results and Discussion

### 2.1 The Preparation of Latex Particles and Latex Films

**FIGURE 1:**
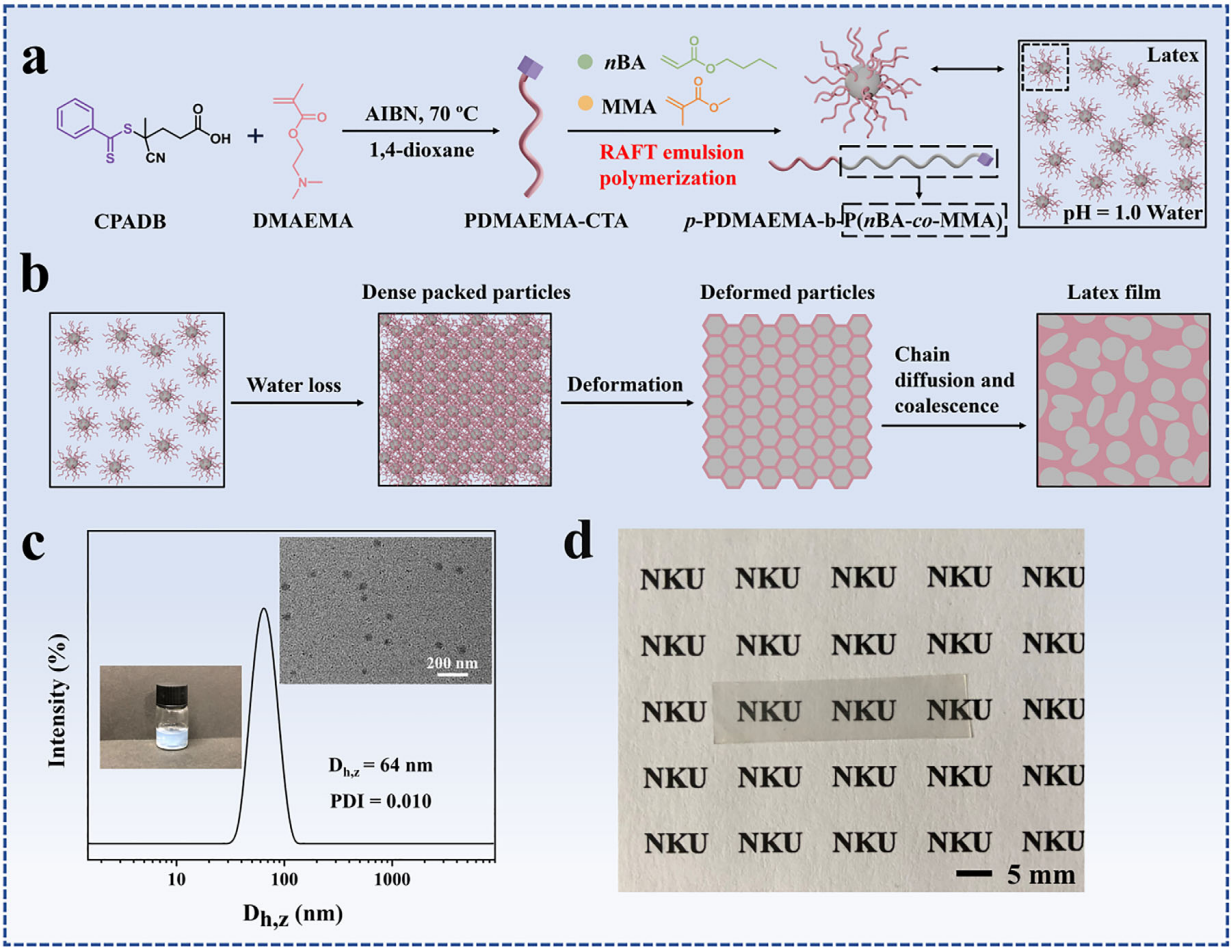
Schematic representations for (a) the synthesis of *p*‐PDMAEMA_207_‐*b*‐P(*n*BA‐*co*‐MMA) latex particles by surfactant‐free reversible addition‐fragmentation chain transfer (RAFT) emulsion polymerization and (b) the film formation of latex particles, (c) dynamic light scattering curve, transmission electron microscopy (TEM) image of latex particles, and a digital photograph of the particle solution, and (d) a photograph of a latex film prepared by casting latex particle solution.

As shown in Figure 1a, surfactant‐free reversible addition‐fragmentation chain transfer (RAFT) emulsion polymerizations of n‐butyl acrylate (*n*BA) and methyl methacrylate (MMA) mediated by protonated poly(2‐(dimethylamino)ethyl methacrylate) (*p*‐PDMAEMA) macro‐chain transfer agent (macro‐CTA) were employed in the synthesis of latex particles with *p*‐PDMAEMA shells and P(*n*BA‐*co*‐MMA) cores. Hydroplastic films were prepared by casting the latex solutions on the pre‐cleaned polytetrafluoroethylene surfaces.

PDMAEMA‐CTA was synthesized by RAFT polymerization. ^1^H NMR spectrum and size exclusion chromatography (SEC) curve of PDMAEMA‐CTA are presented in Figure S1. ^1^H NMR result confirms the synthesis of the macro‐CTA, and the absolute number‐average molecular weight was calculated by SEC equipped with a light scattering detector (SEC‐MALLS). The macro‐CTA used in this research was referred to as PDMAEMA_207_‐CTA, where 207 is the average repeating unit number. In acidic water at pH = 1.0, the tertiary amine groups on the side chains were protonated and water‐soluble *p*‐PDMAEMA_207_‐CTA was obtained.

In previous researches, the mechanism for surfactant‐free RAFT emulsion polymerization mediated by hydrophilic macro‐CTA was studied, and latex particles with different hydrophilic polymers in the coronae and hydrophobic polymers in the cores were synthesized [46, 47, 48]. Herein, surfactant‐free RAFT emulsion copolymerization of *n*BA and MMA mediated by *p*‐PDMAEMA_207_‐CTA was performed to synthesize core‐shell latex particles (Figure 1a). A digital photograph in the inset of Figure 1c indicates that stable bluish‐white solution was obtained after RAFT emulsion polymerization. Dynamic light scattering (DLS) curve and transmission electron microscope (TEM) image of the synthesized latex particles are shown in Figure 1c. DLS result indicates that the average hydrodynamic diameter (D_h,z_) of the particles is 64 nm with a polydispersity index of 0.010, demonstrating the synthesis of monodispersed latex particles. TEM result confirms the synthesis of spherical latex particles with an average size of 42 nm.

After deprotonating *p*‐PDMAEMA_207_‐*b*‐P(*n*BA‐*co*‐MMA) latex particles in a saturated sodium bicarbonate solution, PDMAEMA_207_‐*b*‐P(*n*BA‐*co*‐MMA) block copolymer (BCP) was obtained. Based on ^1^H NMR result, the average repeating unit numbers of *n*BA and MMA were calculated to be 219 and 204, respectively (Figure S2). The volume ratio of the hydrophilic *p*‐PDMAEMA_207_ to hydrophobic P(*n*BA_219_‐*co*‐MMA_204_) component in the latex particles was calculated to be around 1:1.78. The calculation details can be found in the supporting information part. Latex films were prepared by casting latex particle solutions on Teflon surfaces at 40^°^C. A photograph of a latex film is shown in Figure 1d, where a transparent latex film with smooth surface is observed. The transparency of the film suggests that no macrophase separation (domain size > 1 µm) occurs in the latex film [49].

### 2.2 The Phase‐Separation in Latex Film

It is widely recognized that following steps are included in the formation of a latex film from latex particles [50, 51, 52, 53]. As shown in Figure 1b, with the evaporation of water, a dense particle packing is achieved. The latex particles contact each other and water molecules are filled in the interstitial spaces among the particles. Under the capillary pressure, the latex particles deform forming honey‐comb structures and the gaps among the particles are filled by the deformed particles. Finally, polymer chain diffusion at the interfaces results in the disappearance of the particle boundaries and the film formation. In the film, the hydrophilic polymer chains anchored onto the particle surfaces aggregate together forming hydrophilic phases and the polymer chains in the hydrophobic core make diffusion at boundaries forming hydrophobic phases [47, 54]. It is noted that the hydrophilic polymer chains may hinder the diffusion of the core polymer chains due to the incompatibility. In the formed latex films, there are hydrophilic and hydrophobic phases. The unique phase structures endow the latex films with hydroplasticity.

**FIGURE 2:**
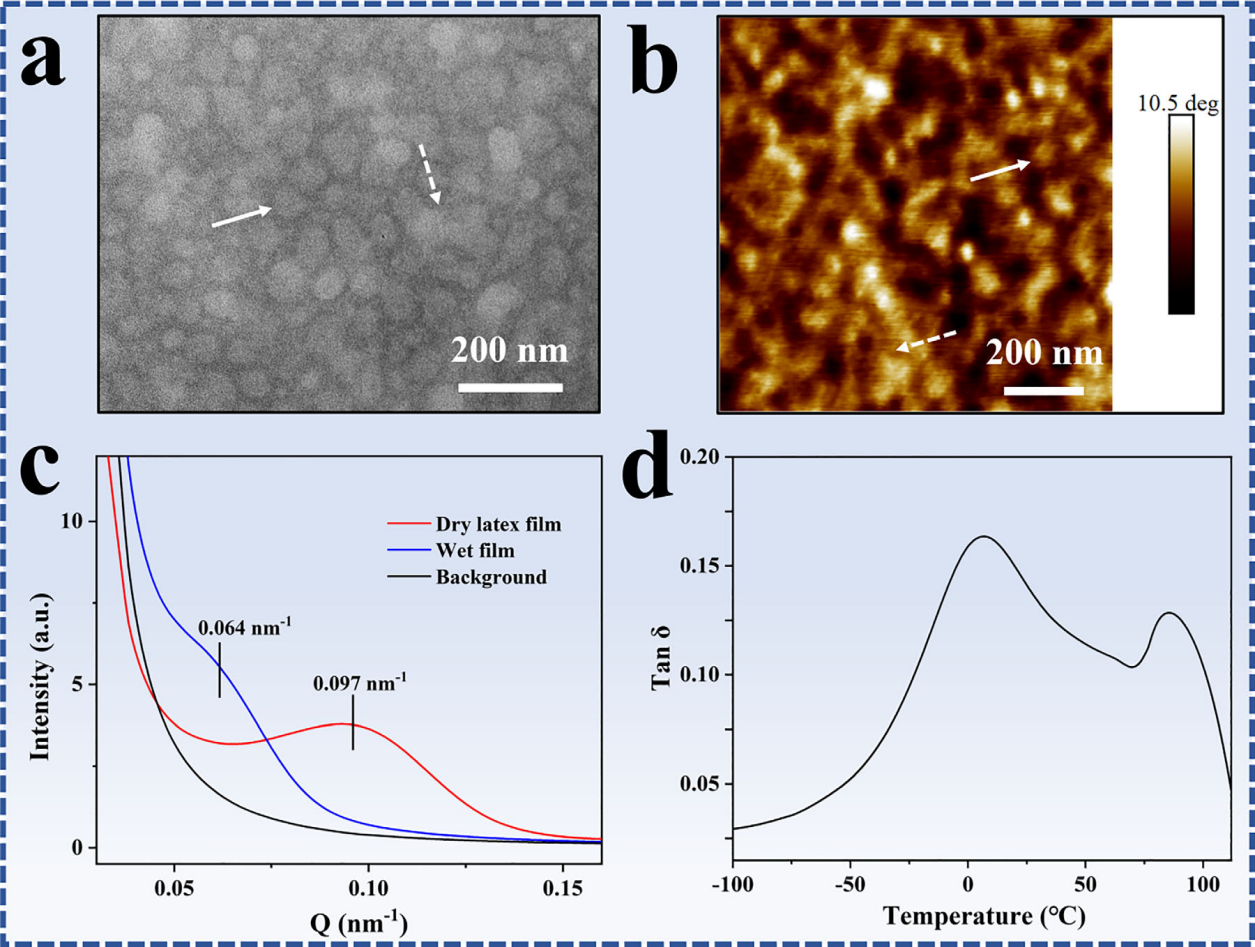
(a) TEM and (b) atomic force microscopy (AFM) image of *p*‐PDMAEMA_207_‐*b*‐P(*n*BA_219_‐*co*‐MMA_204_) latex film, (c) small angle x‐ray scattering (SAXS) curves of the prepared latex film and the wet film after being immersed in water for 50 s, and (d) tan δ curve of the latex film measured by dynamic mechanical analysis (DMA).

A TEM image of latex film formed by casting *p*‐PDMAEMA_207_‐*b*‐P(*n*BA_219_‐*co*‐MMA_204_) latex particles is shown in Figure 2a. The TEM specimen was stained under OsO_4_ atmosphere and the *p*‐PDMAEMA phases were stained. In the image, the dark domains represent the stained *p*‐PDMAEMA phases and the white domains represent P(*n*BA_219_‐*co*‐MMA_204_) phases. The TEM result indicates that in the latex film hydrophilic *p*‐PDMAEMA forms continuous networks that surround the hydrophobic domains. The phase structure in the latex films is dependent on the volume ratio of the hydrophilic to the hydrophobic component, and the length of the hydrophilic block. In a previous research, continuous hydrophobic phases in a latex film were observed due to a high volume percentage of the hydrophobic phases [47]. In addition, the covalently bonded hydrophilic blocks on the particle surfaces restrict the interdiffusion of hydrophobic core blocks in the film formation process [55], and the long hydrophilic blocks are more unfavorable for the hydrophobic chain interdiffusion than the short blocks. Herein, two types of hydrophobic domains, isolated and fused structures, are observed in the TEM image (Figure 2a). The two different structures are indicated by solid and dash arrows, respectively. The size of the isolated hydrophobic domain is around 43.2 nm, which is quite similar to the size of the latex particle, suggesting that some hydrophobic chains do not have chain interdiffusion in the film formation process due to the hindrance of the hydrophilic *p*‐PDMAEMA blocks. Meanwhile, to reduce the interfacial energy between the hydrophilic and the hydrophobic domains the hydrophobic chains in the cores make chain interdiffusion at boundaries, resulting in the formation of the fused hydrophobic cores, as indicated by the dash arrow in Figure 2a. The TEM result is also confirmed by atomic force microscopy (AFM) result. An AFM image of latex film is shown in Figure 2b. The AFM phase image indicates that the latex film possesses a distinct two‐phase structure, the deep domains correspond to the hydrophilic *p*‐PDMAEMA phases and the light domains correspond to the hydrophobic P(*n*BA‐*co*‐MMA) phases [56]. As indicated by a solid and dash arrow, both isolated and fused hydrophobic domains are observed. The size of the isolated structure is around 44 nm, which is close to the sizes of the particle cores determined by TEM.

Small angle x‐ray scattering (SAXS) measurement was employed to analyze the film structure. SAXS reflects electron‐density fluctuations within a sample on the length scale of 1–100 nm. SAXS curve of the latex film is shown in Figure 2c. where a characteristic correlation peak at q ≈ 0.097 nm^−1^ is observed. The reciprocal correlation length is calculated according to the equation d = 2π/q [57], and the calculation result indicates that the correlation length is around 64.7 nm. The measured SAXS signal originates from spatially correlated hydrophobic regions with average electron density different from the surrounding *p*‐PDMAEMA phases [58]. This result confirms the nanosized phase separation in the latex film.

Dynamic mechanical analysis (DMA) was employed to characterize the latex film. The presence of multiple maxima in the mechanical loss tangent is routinely associated with glass transition temperatures (T_g_s) of the polymers in different phases [49]. Tan δ curve of the latex film is shown in Figure 2d. The presence of two broad loss peaks at around 6.4^°^C and 86.7^°^C confirmed phase separation in the latex film. DSC curve of *p‐*PDMAEMA is presented in Figure S3, which indicates that T_g_ of the polymer is at around 19.1^°^C. T_g_s of P*n*BA and PMMA are around −40^°^C and 98^°^C, respectively [59]. So it is reasonable to speculate that the broad peak in the range of −40^°^C–60^°^C is associated with the T_g_s of *p*‐PDMAEMA in the hydrophilic phases, and P*n*BA‐rich domains in the hydrophobic phases, and the peak at 86.7^°^C is associated with the T_g_ of PMMA‐rich domains in the hydrophobic phases. In the hydrophobic phases there are two different domains, P*n*BA‐rich domains and PMMA‐rich domains. The P*n*BA‐rich chains with low T_g_, make chain interdiffusion across the boundaries of the latex particles in the film formation process. Because of the high T_g_, PMMA‐rich domains in the hydrophobic phases serve as physical crosslinking sites at room temperature and endow the latex film with mechanical properties.

### 2.3 The Fabrication of Hydroplastic Latex Film

**FIGURE 3:**
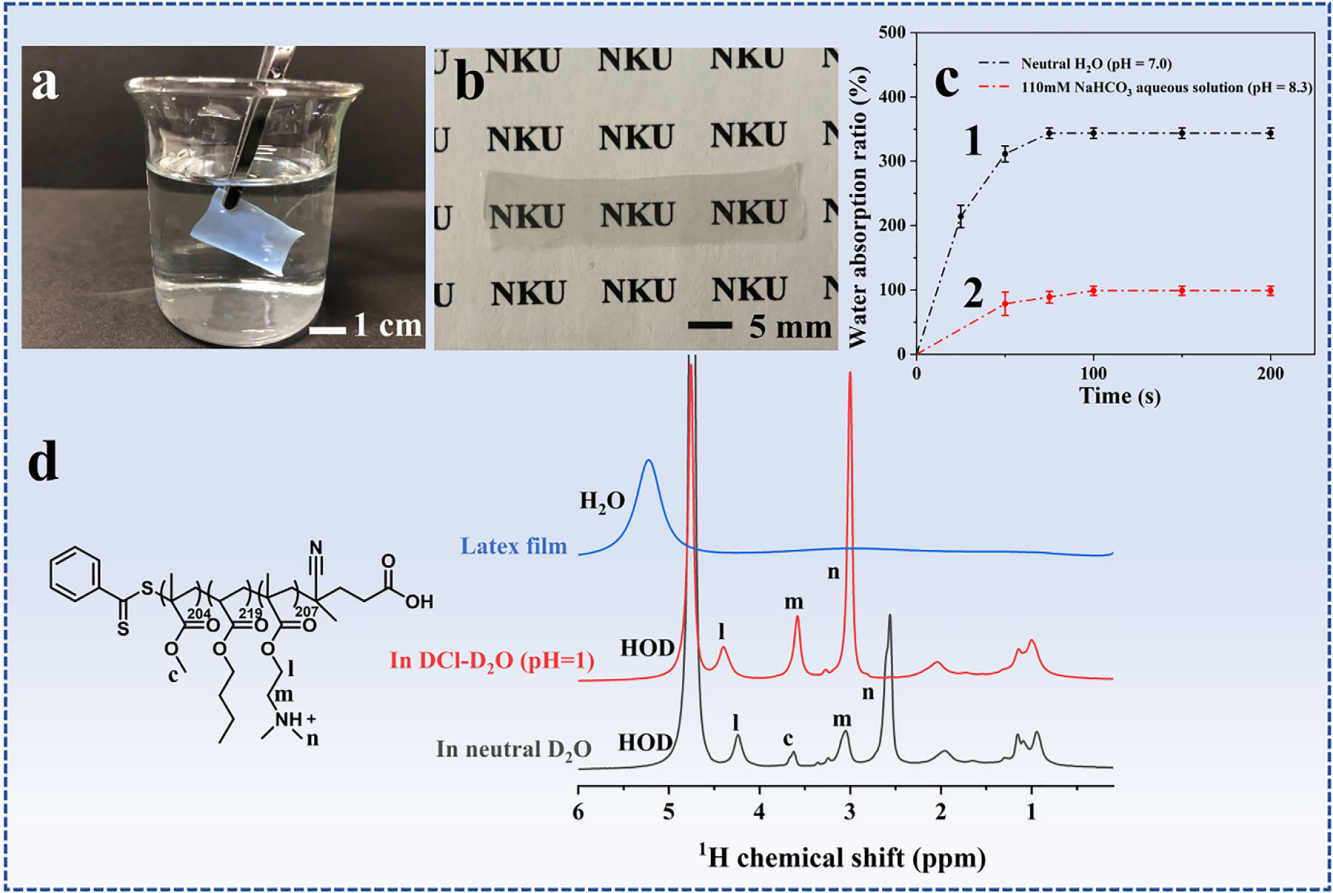
(a) A photograph of latex film immersed in neutral water, (b) photograph of latex film after drying in air, (c) plots of water absorption ratio of latex films in neutral water (pH 7.0) and aqueous solution of NaHCO_3_ (pH 8.3) as a function of immersion time, and (d) ^1^H solid‐state NMR spectra of original latex film, and latex films after being treated in deuterium chloride (DCl)‐D_2_O solution (pH 1.0) and D_2_O.

A photograph of a latex film in neutral water is shown in Figure 3a. Upon immersion of the film in water at 0^°^C, it rapidly changes from transparent to opaque indicative of macro‐phase separation in the film. In water, water molecules diffuse into the hydrophilic *p*‐PDMAEMA phases, which results in the swelling of the hydrophilic phases and a change in the refractive index. After drying in air, water is removed from the *p*‐PDMAEMA phases and the water‐swollen hydrophilic phases shrink, which lead to the recovery of the transparency of the film (Figure 3b).

Curve 1 in Figure 3c shows water absorption ratios of the latex film at different immersion time in neutral water. Herein, water absorption ratio, referred to as the weight ratio of the absorbed water to dry film, is used to measure the water absorption capability of the film. The calculation formula can be found in the supporting information part. The water absorption rate is very high in neutral water. During the first 75 s, the absorption ratio reaches 350 wt.%, and after that point the ratio keeps unchanged indicating the saturation of the latex film. The effect of pH value on the water absorption was investigated. Curve 2 in Figure 3c represents the change of the water absorption ratio after being immersed in aqueous solution of NaHCO_3_ (pH 8.3). The ratio reaches 98% at saturation, much lower than the absorption ratio in neutral water. Upon immersion of the latex film in a base solution, the *p*‐PDMAEMA chains are deprotonated and the formed PDMAEMA is much less hydrophilic than *p*‐PDMAEMA, so the amount of water absorbed in PDMAEMA phases is much less than that absorbed in *p*‐PDMAEMA phases.

Figure S4 shows DSC curves of a latex film after being immersed in neutral water. Before DSC measurement, the latex film was wiped with filter paper and water on the surface was removed. On the cooling curve of the wet latex film, a peak at −12.8^°^C is observed. This peak corresponds to the ice formation in the hydrophilic phases. On the heating curve, a peak at 3.3^°^C is observed, which corresponds to the ice melting. DSC results demonstrate the diffusion of water into the hydrophilic phases.

Solid NMR is sensitive to local chemical environments, and can provide structural details of polymers [60]. Herein, ^1^H solid‐state NMR (^1^H SSNMR) experiments were performed to study the water diffusion into the latex films. Figure 3d shows ^1^H SSNMR spectra of original latex film, films after being treated in D_2_O and deuterium chloride (DCl)‐D_2_O solution (pH 1.0). On the spectrum of the dry latex film, only a peak corresponding to water absorbed on the film surface is observed, and no peaks corresponding to the protons on the polymer are observed due to the low motility of the polymer chains in the dry film. However, after being treated in DCl‐D_2_O, signals corresponding to the methyl and methylene protons on DMAEMA units are observed, demonstrating the diffusion of D_2_O molecules into the hydrophilic *p*‐PDMAEMA domains and an improvement in the chain mobility. The spin‐lattice relaxation time (T_1_) of the protons was obtained. The T_1_ values of the methyl protons (peak n), and methylene protons (peaks m and l) were measured to be 0.451, 0.403, and 0.405 s. On the spectrum of the latex film after being treated in D_2_O, not only the signals corresponding to the protons on DMAEMA units but also a weak signal corresponding to the methyl protons on MMA units is observed (Figure 3d), which indicate that the hydration of *p*‐PDMAEMA domains leads to an improvement in the mobility of PMMA segments at the interfaces of hydrophilic and hydrophobic phases. The T_1_ values of the methyl protons (peak n), and methylene protons (peaks m and l) on DMAEMA units were measured to be 0.419, 0.439 and 0.412 s, and the T_1_ value of the methyl protons on MMA units was 1.57 s.

**FIGURE 4:**
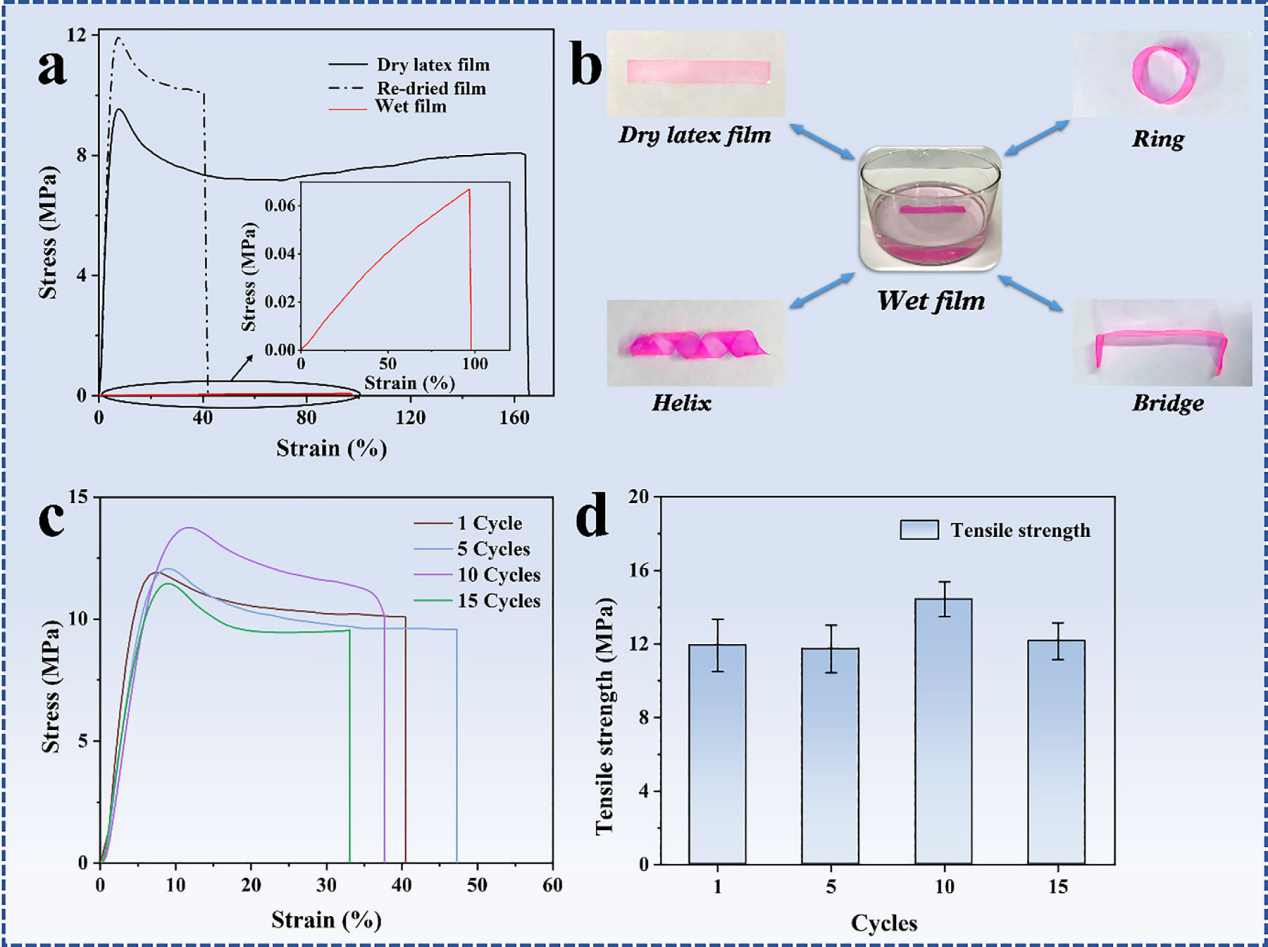
(a) Stress‐strain curves of the original latex film (black), wet film immersed in neutral water at 0^°^C for 50 s (red), and latex film after drying in air (dashed line), (b) illustrations for programming a latex film into diverse 3D shapes by immersing in water at 0^°^C for 50 s, (c) stress‐strain curves of latex films after 1, 5, 10, and 15 wet‐dry cycles, and (d) tensile strengths of latex films after 1, 5, 10, and 15 wet‐dry cycles. In order to observe the latex film clearly in (b), trace amount of rhodamine B was added into the film.

A hydroplastic material with shape processability should have high mechanical properties in dry state, and low mechanical properties in wet state, so that the material can be molded into various shapes after being treated in water. The mechanical properties are illustrated from the tensile tests. The stress–strain curves of a latex film before and after immersion in water are shown in Figure 4a. The original latex film shows a yield strength at 9.6 MPa, a strength‐at‐break at 8.1 MPa and a strain at break at 165%. For the wet latex film, much low mechanical properties are observed. As shown in the inset of Figure 4a, the tensile strength increase linearly with strain until break at 0.07 MPa and 113% strain. Similar to low mechanical properties of thermoplastic polymers in molten state, a low strength stress of a wet film is necessary for shape processability. A high strength stress of a film in wet state makes the processability difficult or impossible. A summary of the mechanical properties of dry and wet latex films is shown in Figure S5. A video showing the shape processability of a wet film is presented in the supporting information part (Video S1).

The hydroplastic processing was performed by immersing latex films in water at 0^°^C for 50 s and programming from a strip to diverse shapes, including bridge, helix, and ring (Figure 4b), and all the shapes can be maintained after drying in air. It is noted that the shaping process is reversible. All the shapes can go back to strip or can be transformed into each other after being immersed in water, which demonstrate the hydroplasticity of the latex films. Stress‐strain curve of a film after drying in air is shown in Figure 4a. The dried film shows yield strength at 11.9 MPa, a strength‐at‐break at 10.1 MPa, and a strain at 42%. It is also noted that the yield strength and strength‐at‐break of the dried film is higher than the original latex film, but the strain is lower. The differences in the mechanical properties are attributed to the phase changes after water treatment.

Herein, the effect of reversible wet‐dry cycles on the hydroplastic latex films is investigated. Dry latex films were immersed in water and mechanical properties were measured after drying. The wet‐dry cycles were repeated for multiple times. The stress‐strain curves of latex films after 1, 5, 10, and 15 wet‐dry cycles are shown in Figure 4c. Based on the stress‐strain curves, the tensile strengths of the films are obtained (Figure 4d). The changes in Young's modulus, breaking elongation, and fracture energy of the latex films after 1, 5, 10, and 15 wet‐dry cycles are shown in Figure S6. It can be observed that the mechanical properties of the latex films after different wet‐dry cycles are very close. This conclusion keeps consistent with the previous literature [32]. Therefore, the mechanical properties of the hydroplastic latex films are stable after multiple wet‐dry cycles.

**FIGURE 5:**
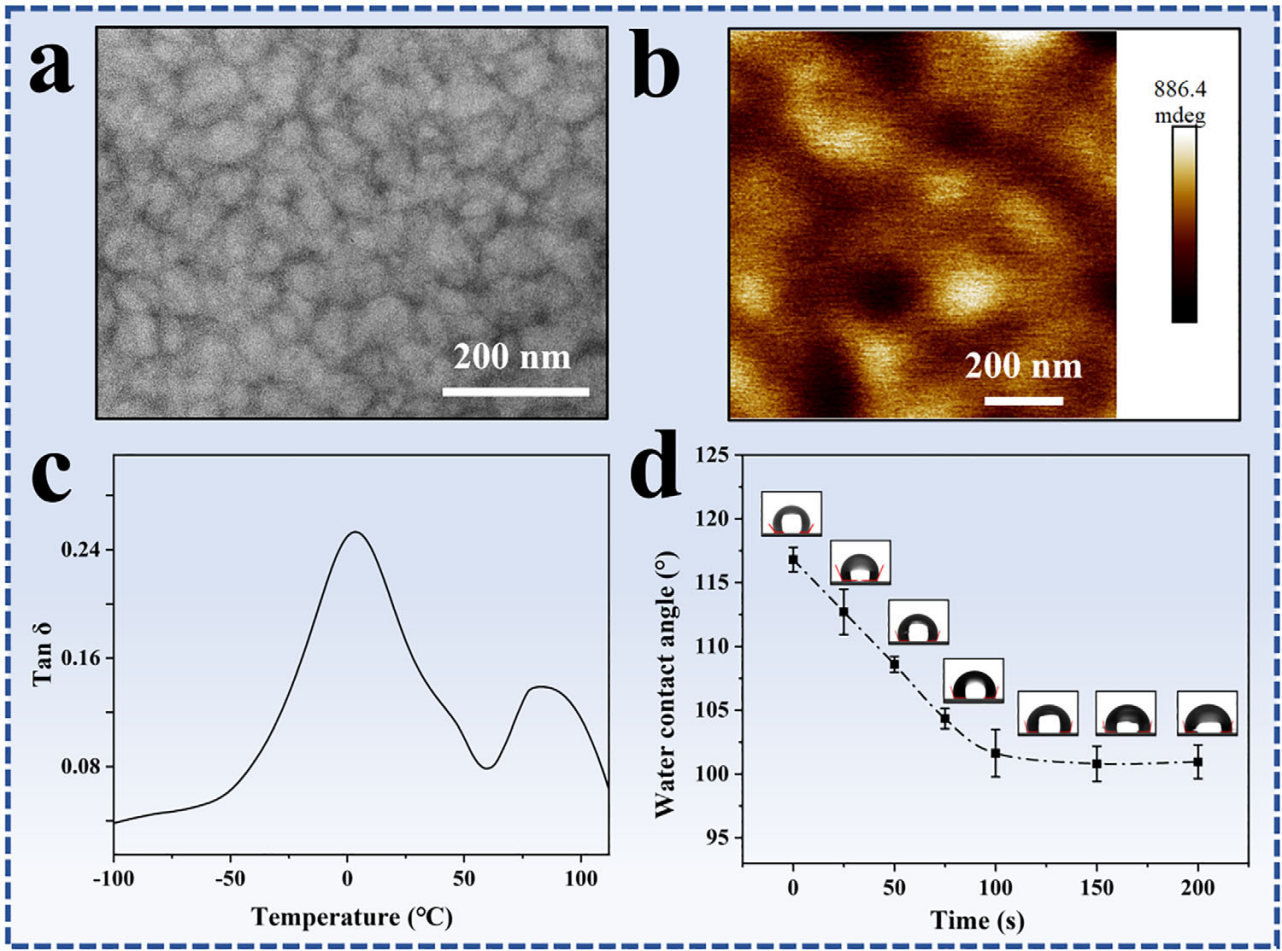
(a) TEM image of the latex film prepared by drying wet film in air, (b) AFM phase image of the wet latex film prepared by immersing the film in water at 0^°^C for 50 s, (c) tan δ curve of the dried latex film, and (d) water contact angles of latex films after immersed in water at 0^°^C for different times.

In thermal processing TPEs have phase structure change upon heating [19, 20, 21, 22, 23, 24, 25]. Similarly, hydroplastic latex films have phase structure change in water treatment. TEM image of a dried latex film after water treatment is shown in Figure 5a. In the TEM image continuous hydrophobic phases formed by fused P(*n*BA‐*co*‐MMA) domains are observed. Comparing with the original latex film (Figure 2a), the sizes of the hydrophobic domains are much bigger. In the casting formation of the film from the latex particles, the hydrophilic *p*‐PDMAMA blocks on the particles hinder the interdiffusion of the hydrophobic polymer chains at the boundaries. However, upon immersion in water, the hydrated *p*‐PDMAEMA exerts much less hindrance on the chain interdiffusion at the boundaries. To reduce the unfavorable interaction between the hydrophobic P(*n*BA‐*co*‐MMA) and the hydrated *p*‐PDMAMA, the hydrophobic chains make boundary interdiffusion, leading to the coalescence of the hydrophobic domains. After the removal of water from the *p*‐PDMAEMA phases, the hydrophilic phases shrink and a dried film with large‐sized hydrophobic phases is formed.

AFM phase image of a wet latex film prepared by immersing the film in water for 50 s, is shown in Figure 5b. Comparing with the original latex film (Figure 2b), the P(*n*BA‐*co*‐MMA) has bigger phase size in wet state, which demonstrates the boundary interdiffusion of the hydrophobic polymer chains and the coalescence of the hydrophobic phases. The sizes of the hydrophilic phases in wet film are much bigger than those in the original film as a result of the swelling of the *p*‐PDMAEMA phases by water. SAXS curve of the wet latex film is presented in Figure 2c. A characteristic correlation peak at q ≈ 0.064 nm^−1^ is observed. As compared to the original latex film (q ≈ 0.097 nm^−1^), the reciprocal correlation length is increased due to the swelling of the *p*‐PDMAEMA phases and the coalescence of the hydrophobic phases. The AFM and SAXS characterizations provide direct proofs on the water‐driven phase separation in the latex film. Tan δ curve of the dried latex film after water treatment is shown in Figure 5c. Similar to the original latex film (Figure 2d), two broad peaks at around 3.5^°^C and 83^°^C are observed. The high T_g_ phases corresponding to the PMMA‐rich domains act as physical crosslinking sites in the water treatment process, and play a vital role in the preparation of the hydroplastic latex films. The effect of water treatment on the phase separation of hydroplastic latex film is similar to the thermal heating on the phase separation of TPEs. Thermal annealing at a temperature above T_g_ of the polymer chains in hard phases promotes phase separation between the soft and hard phases [19, 20, 21, 22, 23, 24, 25]. Water treatment of the hydroplastic latex film allows the coalescence of the hydrophobic phases.

**FIGURE 6:**
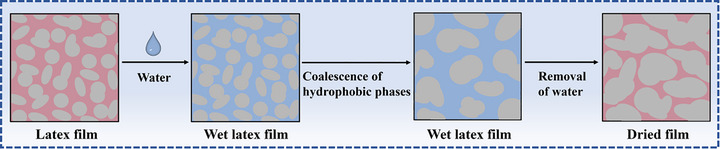
A proposed physical model for water diffusion and water‐driven phase separation in hydroplastic latex film.

A physical model for water diffusion and water‐driven phase separation is presented in Figure 6. Upon immersion of a multi‐phase latex film into water, water molecules diffuse into the hydrophilic phases, leading to the swelling of the phases. Because of the incompability between hydrated *p*‐PDMAEMA and P(*n*BA‐*co*‐MMA), the hydrophobic polymer chains make boundary interdiffusion, leading to the coalescence of the hydrophobic domains so that the interfacial energy between hydrophilic and hydrophobic phases can be reduced. Meanwhile, swelling of the hydrophilic phases results in a decrease in the viscosity, which is kinetically favorable for the hydrophobic phase coalescence. After the removal of water, latex films with large‐sized hydrophobic phases are formed.

In a previous research, we studied the wetting behaviors of the films formed by casting solutions of latex particles with poly(*n*‐butyl methacrylate) (P*n*BMA) cores and zwitterionic coronae [47]. The latex films had dynamic surfaces. Water treatment exerted a significant effect on the wetting behaviors. Because of an increase in the hydrophilicity of latex film, the water contact angle decreased with water‐treatment time. Herein, the influence of water treatment on the water contact angle of the hydroplastic film is investigated. The hydroplastic film was immersed in water for different times at 0^°^C, and after wiping water on the film with filter paper the water contact angles were measured. As shown in Figure 5d, the water contact angle is negatively proportional to the water treatment time. In the casting formation of the latex film in air, driven by the reduction of surface‐energy the hydrophobic polymer chains tend to occupy the film surface [61]. Upon immersion of the latex film in water, water molecules diffuse into the hydrophilic *p*‐PDMAEMA phases, and the hydrated polymer chains migrate to the film surface due to the hydrophilic medium, which results in an increase in the surface hydrophilicity and a decrease in the water contact angle.

## 3 Conclusion

In conclusion, latex particles with hydrophobic cores and hydrophilic shells were synthesized by RAFT emulsion polymerization. Latex films with hydrophobic P(*n*BA‐*co*‐MMA) phases surrounded by continuous hydrophilic *p*‐PDMAEMA phases, were prepared by casting latex particle solution. Upon immersion in water, the latex films have water‐driven phase separation. Water molecules diffuse into the hydrophilic phases, resulting in swelling of the hydrophilic phases and the interdiffusion of P(*n*BA‐*co*‐MMA) chains at the boundaries between the hydrophilic and hydrophobic phases. Water treatment leads to a decrease in the mechanical properties of the films, which allows molding of the latex films into various shapes at room temperature. The different shapes can be fixed in dry state due to the existance of the high T_g_ domains in the hydrophobic phases. This research provides an unique approach to the synthesis of hydroplastic materials, and it is expected that more functional hydroplastics can be prepared by using this eco‐friendly and low‐cost method.

## Funding

The National Natural Science Foundation of China (NSFC, 22471135)

## Conflicts of Interest

The authors declare no conflicts of interest.

## Supporting information




**Supporting File 1**: advs73574‐sup‐0001‐SuppMat.docx.

**Supporting File 2**: advs73574‐sup‐0002‐VideoS1.mp4.

## Data Availability

The data that support the findings of this study are available from the corresponding author upon reasonable request.;
