# Supplementary material for: Reprogrammable Hydroplastic Latex Films and Water‐Driven Phase Separation
Source: Adv Sci (Weinh). 2025 Dec 27;13(14):e23178. doi: 10.1002/advs.202523178 (PMC12970186; doi:10.1002/advs.202523178)
Supplement: Supplementary file 1 — Supporting File 1: advs73574‐sup‐0001‐SuppMat.docx. [file ADVS-13-e23178-s001.docx]

Supporting Information

**Reprogrammable Hydroplastic Latex Films and Water-Driven Phase Separation**

*Yuwei Du, and Hanying Zhao**

**CONTENTS**

[**1.** **Materials 22**](#_Toc208606954)

[**2.** **Characterization 2**](#_Toc208606955)**2**

[**3.** **Synthesis of PDMAEMA_207_-CTA 2**](#_Toc208606956)**3**

[**4.** **RAFT-mediated emulsion copolymerization of *n*BA and MMA 2**](#_Toc208606957)**4**

[**5.** **Preparation of latex film from latex particles 2**](#_Toc208606958)**4**

[**6.** **The volume ratio of the hydrophilic to hydrophobic component in latex particles 2**](#_Toc208606959)**4**

[**7.** **The calculation of water absorption ratios of the latex film 2**](#_Toc208606960)**4**

[**References 2**](#_Toc208606961)**5**

[**Supplementary Figures 2**](#_Toc208606962)**6**

1. **Materials**

2-(Dimethylamino)ethyl methacrylate (DMAEMA, 99%, Sigma-Aldrich), n-butyl acrylate (nBA, 99%, Macklin), and methyl methacrylate (MMA, 99%, Sigma-Aldrich) were purified by passing through basic alumina columns. 2,2’-Azoisobutyronitrile (AIBN, 97%, Sinopharm Chemical Reagent Co., Ltd.) was purified by recrystallization from ethanol. (4-Cyanopentanoic acid) dithiobenzoate (CPADB) was synthesized in this laboratory.[1] 2,2'-Azobis[2-(2-imidazolin-2-yl)propane] dihydrochloride (VA-044, 98%, Heowns) was used as received. 1,4-Dioxane (AR, Tianjin guangda Chem.) was distilled before use.

1. **Characterization**

^1^H NMR spectra were collected on a Bruker Avance III 400 MHz spectrometer using deuterated chloroform as solvents. *p*-PDMAEMA_207_-*b*-P(*n*BA_219_-*co*-MMA_204_) latex particles were deprotonated in a saturated sodium bicarbonate solution. After dialysis against water and freeze-drying, ^1^H NMR measurement of the block copolymer (BCP) was carried out.

Solid-state NMR experiments were performed on a Bruker AVANCE NEO wide-bor (89 mm) NMR spectrometer operating at proton frequencies of 400.18 MHz. A conventional 4 mm double-resonance MASDVT400W1 probe was used, and the 90° pulse width was 2.5 μs on ^1^H RF channels. Magic angle spinning (MAS) was automatically controlled at 5 kHz within ± 1 Hz with a MAS speed controller.The spin-lattice relaxation time (T1) of proton was measured by inversion-recovery pulse sequence. The recycle delay was set to 3 s. The ^1^H chemical shifts was referenced to external TMS (0 ppm).

The absolute molecular weights and distributions of the polymers were characterized on a size exclusion chromatography (SEC) system equipped with a light scattering detector (SEC-MALLS), and DMF was used as the mobile phase. The details about the equipment can be found in our previous publications.^[2,3]^

Z-average sizes (D_h,z_) and size distributions of the latex particles were obtained on a Malvern Zetasizer Nano-S90 equipped with a 10 mW He-Ne laser (633 nm) at an angle of 90°.

Transmission electron microscopy (TEM) images were obtained on a HITACHI HT7700 transmission electron microscope, operated at a voltage of 100 kV. The TEM specimens of the latex particles were prepared by using the freeze-drying method. Latex particle solution was diluted with water, and the diluted solution was dripped onto a Formvar-coated copper grids. After the removal of the excess liquid with filter paper, the copper grid was promptly transferred to a stage immersed in liquid nitrogen for rapid freezing. TEM specimen was obtained after vacuum freeze-drying. The TEM specimen of a latex film was prepared by casting diluted latex particle solution on a Formvar-coated copper grid at room temperature, and the solvent was evaporates in air. All the TEM specimens were stained under OsO_4_ atmosphere for 1 h.

Differential scanning calorimetry (DSC) measurements were conducted on a Mettler-Toledo DSC822 under a nitrogen atmosphere at the scanning rate of 10 K·min^-1^.

Water contact angle was measured on a contact angle meter (JC2000D1, Zhongchen Digital Tech) at room temperature. One drop of water (about 2 μL) was dropped on the surface of the latex film. It was photographed with a CCD camera and the contact angle was calculated. Dry latex film sample was prepared by casting latex particle solutions onto a 2 cm × 2 cm glass slide and drying in an oven at 40 °C. After drying, the coated latex film was annealed in an oven at 120 °C for 12 h. To investigate the effect of water treatment on the contact angle, the latex film was immersed in water at 0 ^°^C for different time, and the contact angles were measured immediately after excess water on the surface was removed using filter paper.

Dynamic mechanical analysis (DMA) measurements were performed on a dynamic mechanical analyzer (DMA 242, Netzsch Mettler-Toledo) with stretching mode at a heating rate of 10 K·min^-1^ and a frequency of 1 Hz. The stress-strain curves were obtained on a Instron 3365 uniaxial testing machine at a tensile loading rate of 100 mm·min^-1^ at 25 °C and 25 % humidity.

Atomic force microscopy (AFM) images were collected on a Dimension Icon AFM operated in the tapping mode under ambient conditions. The scanning range in the Z direction is 8 μm, and the range in the X-Y direction is 1 μm × 1 μm.

Small angle X-ray scattering (SAXS) results were collected on a small angle X-ray scattering instrument (Xenocs Xeuss 3.0).

1. **Synthesis of PDMAEMA_207_-CTA**

PDMAEMA_207_-CTA was synthesized by CPADB mediated RAFT polymerization of DMAEMA. DMAEMA (7.08 g, 45.0 mmol), AIBN (3.9 mg, 0.024 mmol), and CPADB (44.8 mg, 0.161 mmol) were dissolved in 7 mL of 1,4-dioxane in a 50 mL Schlenk flask. After three freeze-pump-thaw cycles, RAFT polymerization of DMAEMA was carried out at 70 ^°^C for 10 h. After the polymerization, the macro-CTA was precipitated in hexane, and dried under reduced pressure at room temperature.

1. **RAFT-mediated emulsion copolymerization of *n*BA and MMA**

RAFT emulsion copolymerization of *n*BA and MMA mediated by PDMAEMA_207_-CTA was described as follows. PDMAEMA_207_-CTA (80.0 mg, 2.44 μmol), VA-044 (0.790 mg, 2.44 μmol), *n*BA (72 mg, 0.56 mmol), MMA (48 mg, 0.48 mmol) were dissolved/dispersed in 3.5 mL of ultrapure water (pH = 1.0) in a 10 mL Schlenk flask. After three freeze-pump-thaw cycles, the mixture was ultrasonically dispersed for 1 min at room temperature. The emulsion polymerization was carried out at 40 °C. After the polymerization, the flask was immersed in ice water and the solution was exposed to the atmosphere.

1. **Preparation of latex film from latex particles**

A transparent latex film was obtained by casting 1.4 mL of latex particle solution onto a polytetrafluoroethylene mold (10 × 50 × 1 mm) and drying in an oven at 40 ^°^C for 6 h. In order to observe the latex film clearly, 14.9 μL of aqueous solution of Rhodamine B (20 mg/mL) was added into the particle solution. The obtained latex film was annealed in a vacuum oven at 120 ^°^C for 12 h.

1. **The volume ratio of the hydrophilic to hydrophobic component in latex particles**

The volume ratio is calculated using the following equation,

$$\begin{aligned} r=\frac{\frac{{207\times M}_{DMAEMA}}{\rho_{PDMAEMA}}}{\frac{219\times M_{nBA}}{\rho_{PnBA}}+\frac{204\times M_{MMA}}{\rho_{PMMA}}} \end{aligned}$$

where $r$ is the volume ratio, $M$ is the molecular weight of the repeating units, and $\rho$ is the polymer density.

The densities of PMMA, P*n*BA, and PDMAEMA are 1.188^[4]^, 1.087^[5]^, and 1.35^[6]^ g/cm^3^, respectively. According to the above equation, the volume ratio of the hydrophilic to hydrophobic component in *p*-PDMAEMA_207_-b-P(*n*BA_219_-*co*-MMA_204_) latex particles is calculated to be 1:1.78.

1. **The calculation of water absorption ratios of the latex film**

Water absorption ratio of the latex film was calculated using the following formula:

$$\begin{aligned} Water absorption ratio=\frac{m_{absorbed water}}{m_{dry film}}\times100\%=\left[ \frac{\left( m_{wet film}-m_{dry film} \right)}{m_{dry film}} \right]\times100\% \end{aligned}$$

Where $m_{absorbed water}$ is the weight of water absorbed by the latex film, $m_{dry film}$ is the weight of the latex film, and $m_{wet film}$ is the weight of the latex film after being immersed in water.

**References**

[1] X. Zhang, X. Lian, L. Liu, J. Zhang, H. Zhao, *Macromolecules* **2008**, *41*, 7863.

[2] C. Wang, J. Zhang, H. Zhao, *J Polym Sci* **2023**, *61*, 3055.

[3] L. Wang, L. Liu, H. Zhao, *Angew Chem Int Ed* **2023**, *62*, e202304073.

[4] J. Brandrup, E. H. Immergut, E. A. Grulke, *Polymer Handbook*, Wiley, New York, **1999**.

[5] R. V. Godbole, F. Khabaz, R. Khare, R. C. Hedden, *J. Phys. Chem. B* **2017**, *121*, 7963.

[6] A. Zengin, G. Karakose, T. Caykara, *Eur. Polym. J* **2013**, *49*, 3350.

**Supplementary Figures**


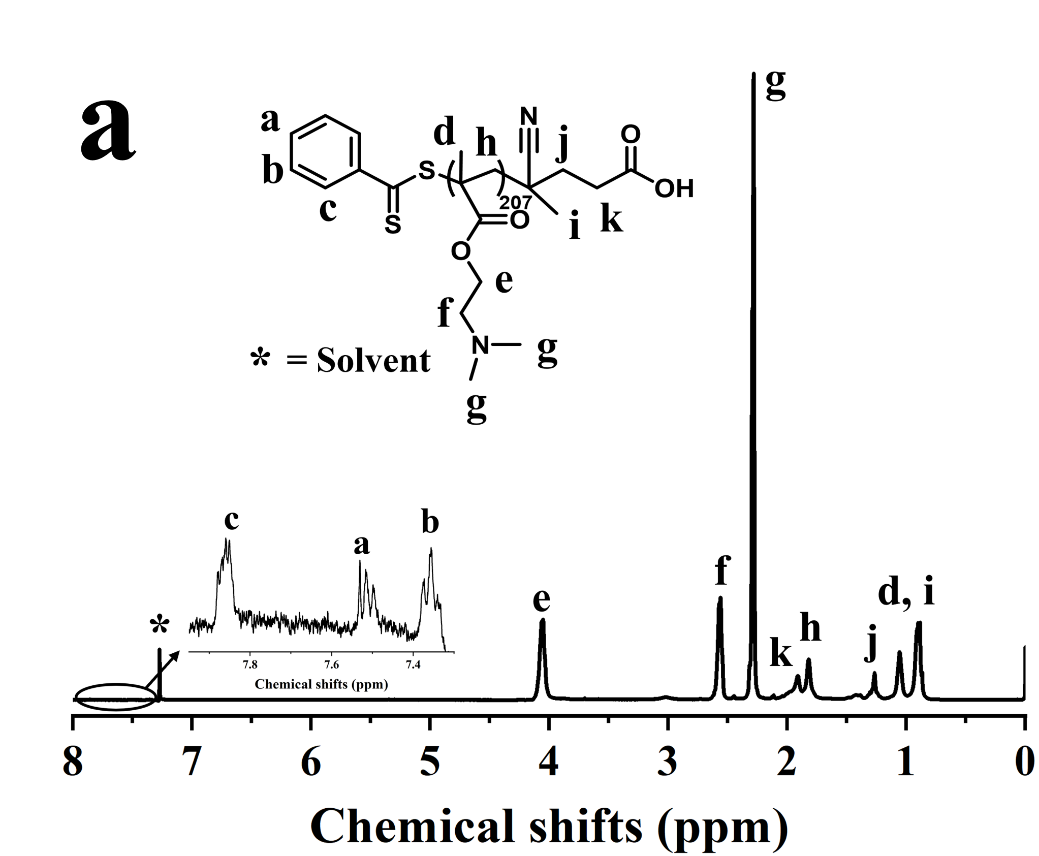


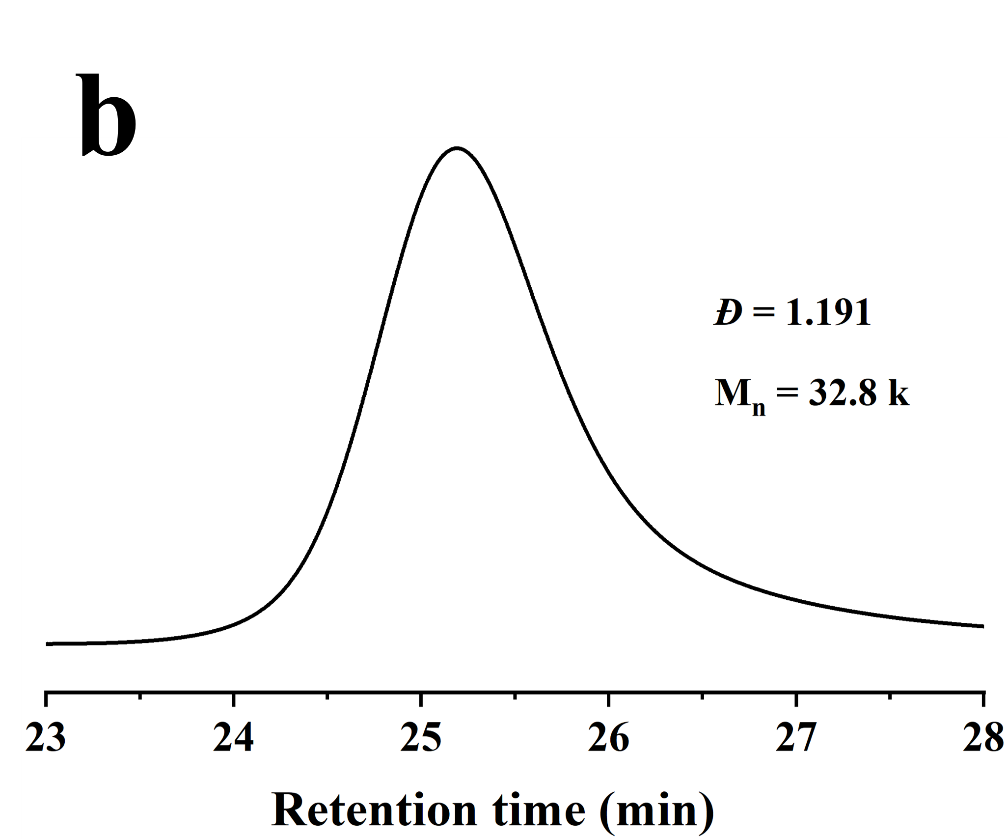


**Figure S1.** (a) ^1^H NMR and (b) SEC curve of PDMAEMA-CTA.


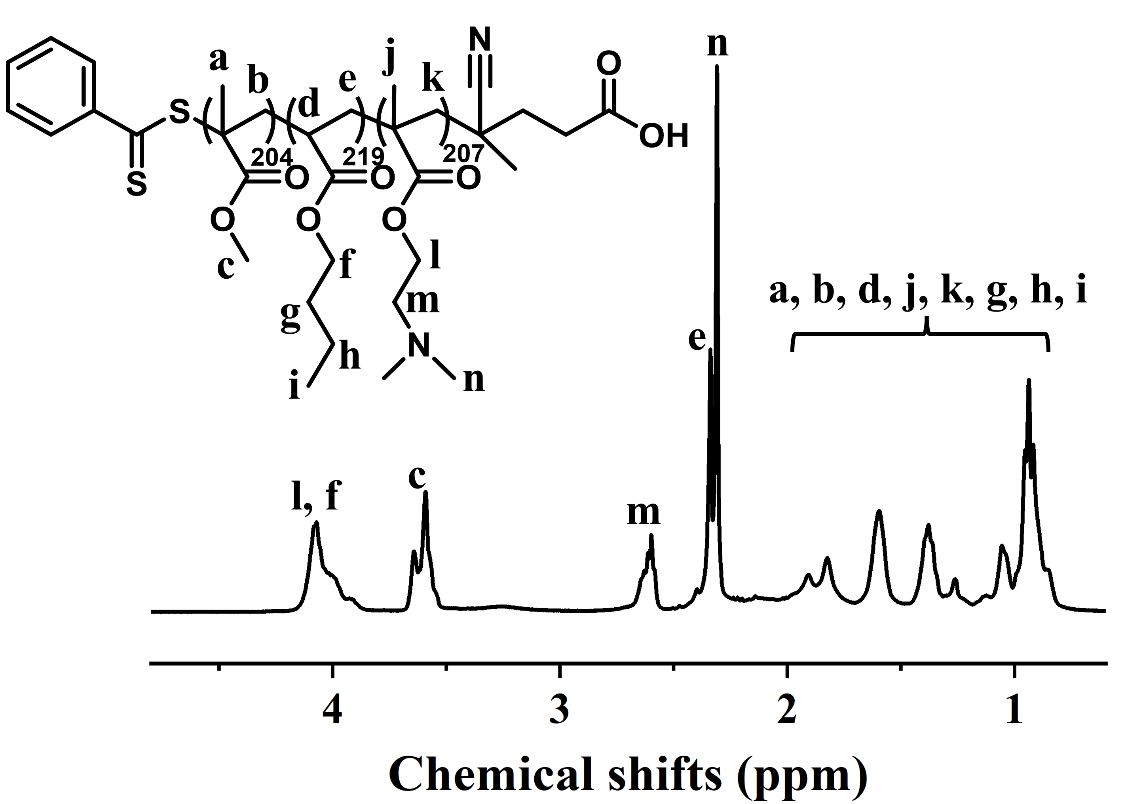


**Figure S2.** ^1^H NMR of PDMAEMA_207_-*b*-P(*n*BA-*co*-MMA) block copolymer.


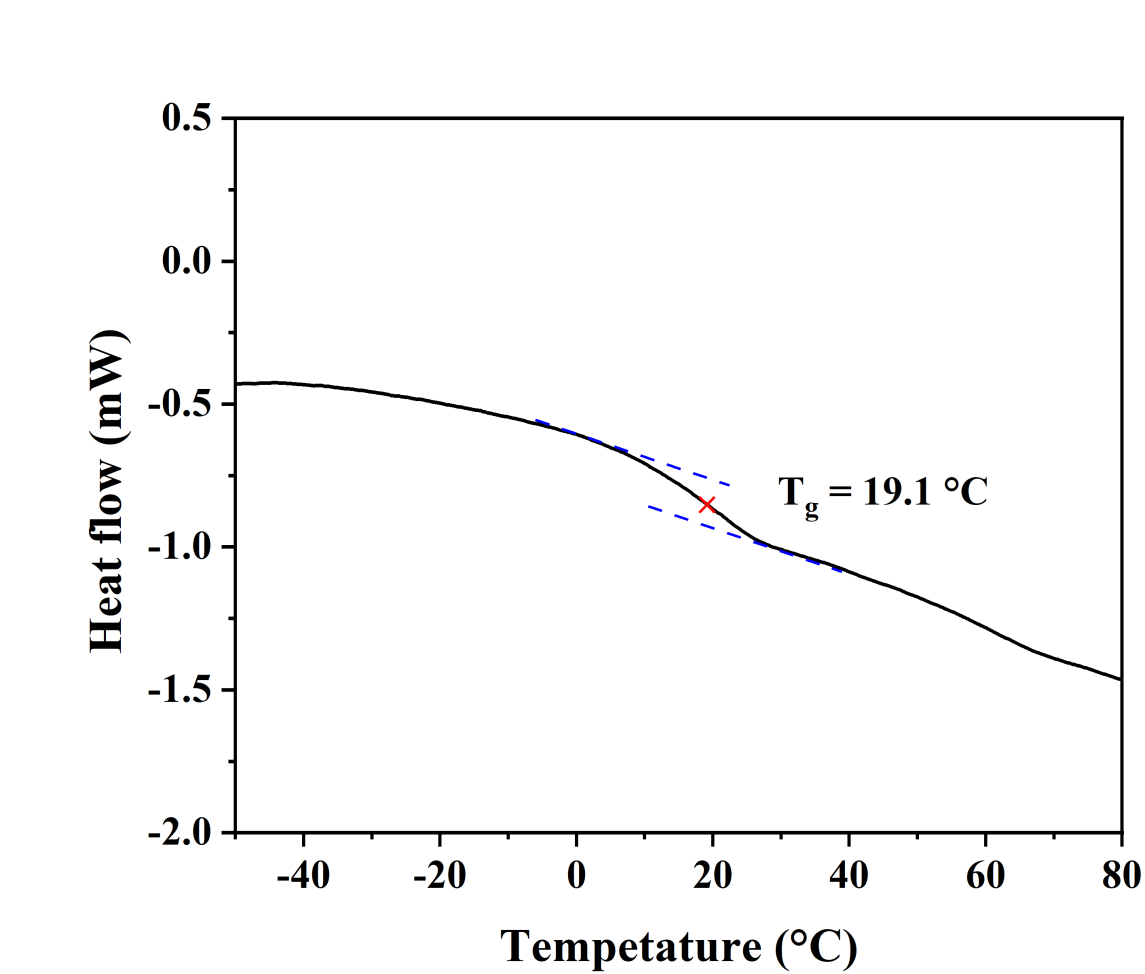


**Figure S3.** DSC curve of *p*-PDMAEMA_207_.


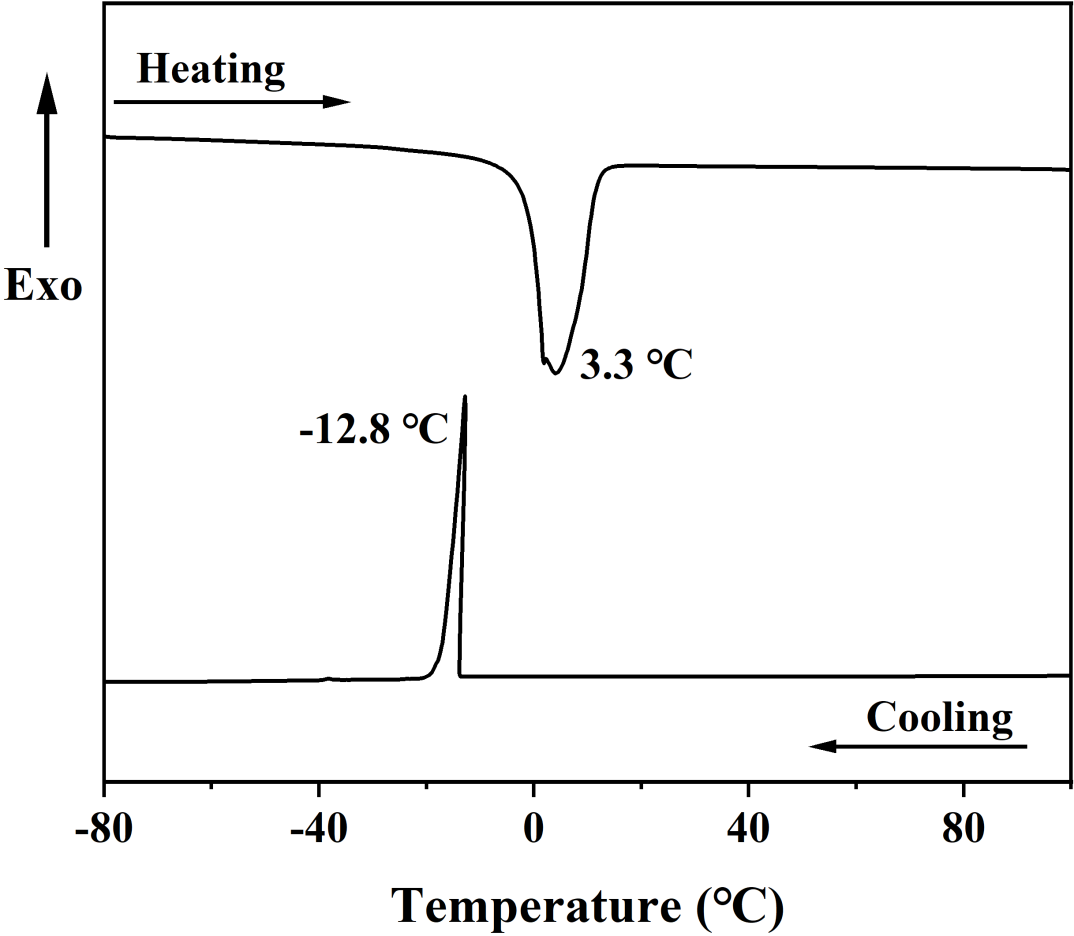


**Figure S4.** DSC cooling and heating curves of a latex film after being immersed in water.


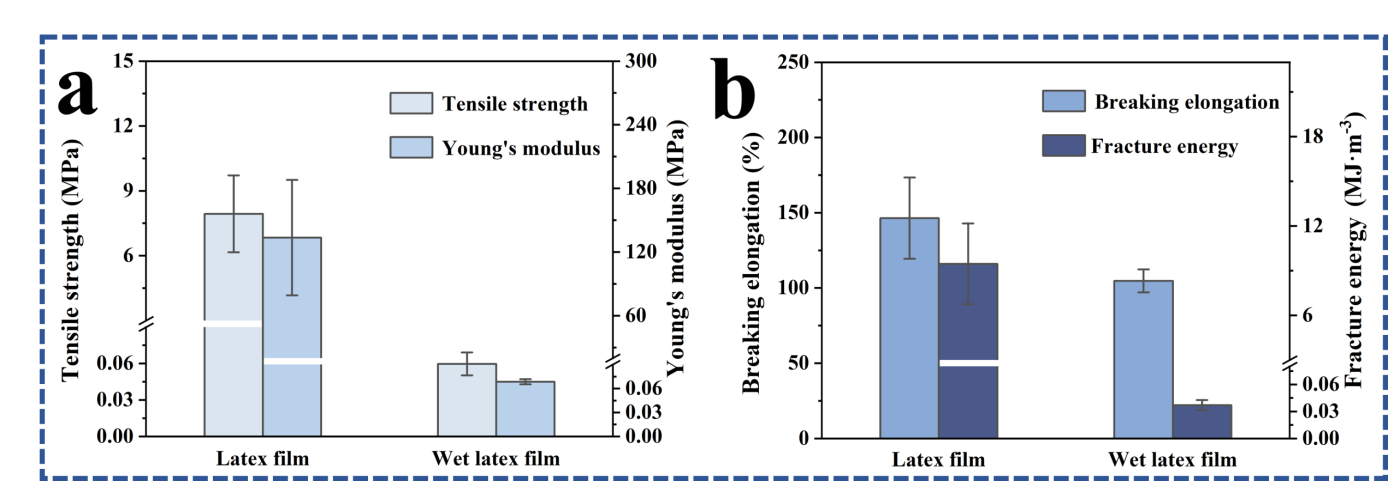


**Figure S5**. Summary of (a) tensile strength and Young’s modulus, and (b) breaking elongation and fracture energy of original and wet latex films.


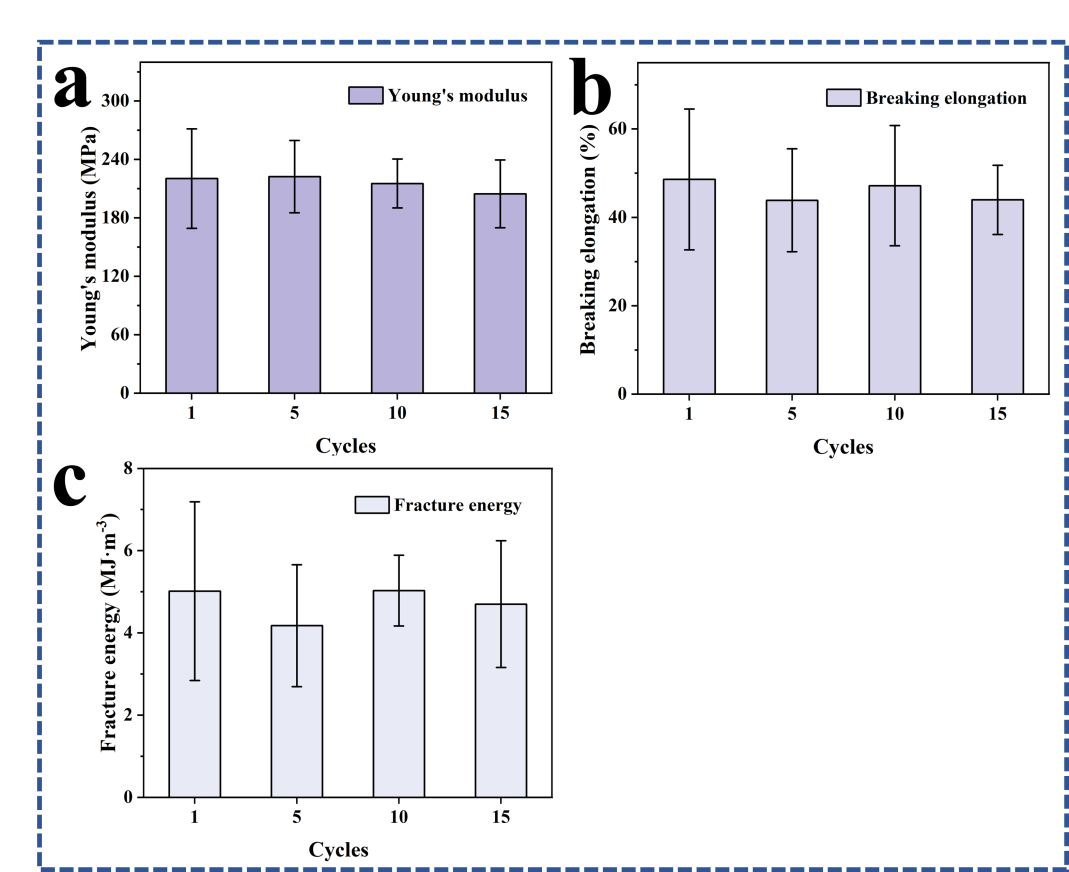


**Figure S6.** The changes in (a) Young’s modulus, (b) breaking elongation and (c) fracture energy of latex films after 1, 5, 10 and 15 wet-dry cycles.
